# Buspirone Hydrochloride Polymorphism: Stability, Solubility, Flow Properties, and Manufacturing Implications

**DOI:** 10.1155/adpp/1941957

**Published:** 2025-10-17

**Authors:** Jéssika Adriane Janning, Victória Guimarães Santiago, Ederlan de Souza Ferreira, Carolina Oliveira de Souza, Márcia Nunes da Silva, Filipa Sofia Reis, Nelson Barros Colauto, Renato Eising

**Affiliations:** ^1^ Graduate Program in Bioscience Technologies, Federal University of Technology-Paraná, Toledo, Brazil, utfpr.edu.br; ^2^ Graduate Program in Food Science, Federal University of Bahia, Salvador, Brazil, ufba.br; ^3^ Graduate Program in Pharmaceutical Sciences, State University of Maringá, Maringá, Paraná, Brazil, uem.br; ^4^ Montain Research Center, Polytechnic Institute of Bragança, Santa Apolónia, Bragança, Portugal, ipb.pt; ^5^ Associated Laboratory for Sustainability Associado and Technology in Inland Regions, Polytechnic Institute of Bragança, Santa Apolónia, Bragança, Portugal, ipb.pt

**Keywords:** buspirone hydrochloride polymorphism, flow property, pharmaceutical formulation development, solubility, stability

## Abstract

Buspirone hydrochloride, an anxiolytic agent, has two interconvertible polymorphic forms that may affect its physicochemical properties and therapeutic efficacy. Despite this relevance, polymorphism is often neglected in pharmaceutical analyses, potentially leading to inconsistent results and compromised drug performance. This study investigates the polymorphism, stability, solubility, and flow properties of commercial samples of buspirone hydrochloride, focusing on how polymorphic transformations affect pharmaceutical performance. Samples from two international suppliers were stored under controlled and stress conditions (humidity and temperature) in open and closed vials. Structural characterization used Fourier‐transform infrared spectroscopy, differential scanning calorimetry, and powder X‐ray diffraction, while flow and density properties were determined by Carr index and Hausner ratio. Solubility of pure polymorphic Forms 1 and 2 was evaluated in physiologically relevant pH media using high‐performance liquid chromatography. The Indian sample contained a mixture of Forms 1 and 2, whereas the Finnish sample consisted exclusively of Form 2. Under stress, Form 2 converted to Form 1, completely in open vials and partially in closed ones, confirming the greater thermodynamic stability of Form 1. Among the analytical methods, differential scanning calorimetry proved to be the most effective in distinguishing polymorphic forms and identifying mixtures. Both forms showed pH‐dependent solubility, with peak dissolution at pH 1.2, and very poor flow properties, requiring formulation adjustments. Solubility data supported a preliminary classification of both polymorphs as Biopharmaceutics Classification System Class I or Class III, although permeability differences remain unexplored. These findings advance the understanding of buspirone hydrochloride’s polymorphic properties, supporting the development of more effective formulations.

## 1. Introduction

The pharmaceutical industry consistently faces the challenge of ensuring uniform quality and performance of drug products. Polymorphism, the phenomenon where a compound can exist in multiple crystalline forms, introduces variability in critical drug properties such as solubility and dissolution rate, which may lead to unpredictable drug delivery and diminished efficacy. Addressing these issues requires a deep understanding of polymorphism and its effects on drug formulation [1]. Therefore, a comprehensive knowledge of polymorphism is fundamental to successfully developing and commercializing pharmaceutical products.

Polymorphism presents substantial obstacles for the pharmaceutical industry, as differences in solid‐state properties can significantly affect drug performance. Ritonavir, for example, was withdrawn from the market due to bioavailability issues stemming from its polymorphic form [2]. Similarly, rotigotine and chloramphenicol palmitate underwent challenges from polymorphic transitions that impacted their solubility, thus reducing therapeutic efficacy [2, 3]. Consequently, the variability introduced by polymorphism can decrease solubility and therapeutic effectiveness and, in severe cases, may lead to market withdrawal of the affected products.

Polymorphic transformations can occur during manufacturing or storage, raising stability concerns. Moisture exposure, for example, can trigger hydrate formation in drugs such as fluconazole, which reduces solubility and complicates the stability profile [2]. To address these risks, regulatory agencies such as the Food and Drug Administration (FDA) in the United States and the National Health Surveillance Agency (ANVISA) in Brazil require detailed documentation on polymorphism during drug registration to ensure product safety and efficacy [4–6].

To thoroughly analyze polymorphism, advanced analytical techniques are employed, including thermal methods such as thermogravimetry and differential scanning calorimetry (DSC), as well as Fourier‐transform infrared (FTIR) spectroscopy, single‐crystal and powder X‐ray diffraction (XRD), microscopy, and nuclear magnetic resonance. These methods allow for precise characterization of polymorphic forms, providing essential data to support the development of stable and effective pharmaceutical formulations [1].

Buspirone hydrochloride, an anxiolytic drug, acts on serotonin, dopamine, and adrenaline receptors and has shown potential for treating various conditions, including generalized anxiety disorder, depression, and cognitive impairments [7]. Two polymorphs of buspirone hydrochloride, Form P188 and Form P203 [8, 9], also referred to as Form 1 and Form 2 [10, 11], have been identified, with reports of interconversion between these forms during storage [8]. This interconversion could impact the drug’s stability and efficacy, as changes in polymorphic structure can affect solubility, bioavailability, and shelf life, potentially compromising therapeutic effectiveness. Despite these implications, specific research on the effects of polymorphism in buspirone hydrochloride remains limited, and no standardized polymorphic form has been established for commercial production. Consequently, buspirone hydrochloride is classified as a high‐solubility, high‐permeability drug (Class I) under the Biopharmaceutical Classification System (BCS) [12], regardless of its polymorphic form, with the impact of polymorphism remaining understudied. Therefore, this study aims to assess the polymorphism, stability, solubility, and flow properties of commercial samples of buspirone hydrochloride to enhance pharmaceutical formulation processes.

## 2. Materials and Methods

### 2.1. Materials

Commercial samples of buspirone hydrochloride, with the chemical formula C_21_H_32_ClN_5_O_2_ (422.0 g mol^−1^) and the IUPAC name 8‐[4‐(4‐(pyrimidin‐2‐yl)piperazin‐1‐yl)butyl]‐8‐azaspiro[4.5]decane‐7,9‐dione; hydrochloride, were provided by the pharmaceutical company Prati‐Donaduzzi, located in Toledo, Paraná, Brazil. These samples were obtained from two raw material suppliers: one in India, labeled as Sample I, and the other in Finland, labeled as Sample II. The suppliers’ names have been omitted to maintain impartiality and keep the study focused. The sample donation was independent of financial support from Prati‐Donaduzzi or other companies.

### 2.2. Sample Preparation

The samples were divided into three groups: (A) Control sample, as received by the supplier; (B) sample stored in amber glass vials with open lids and placed in a climate chamber (Mecalor, model EC/1,2 AR‐URC) at 75% relative humidity and 50°C for 48 days; and (C) sample stored in amber glass vials with closed lids, kept under the same conditions as Group B. Each sample was identified by supplier origin, Sample I or Sample II, and labeled according to the applied conditions: control (IA or IIA), degradation induction in an open vial (IB or IIB), and degradation induction in a closed vial (IC or IIC). After this period, the samples underwent a comprehensive physicochemical analysis of their structural, thermal, and molecular properties.

### 2.3. FTIR Spectroscopy Characterization

The buspirone hydrochloride samples were analyzed via FTIR spectroscopy using a spectrometer (PerkinElmer, Spectrum Frontier) equipped with a universal attenuated total reflection (ATR) sampling accessory. Each spectrum was recorded at a resolution of 2 cm^−1^, with 16 scans conducted over the 4000–650 cm^−1^ range. Data processing was performed using the Spectrum ES software (PerkinElmer Inc.). Analyses were conducted directly on the samples without any pretreatment.

### 2.4. DSC Characterization

Buspirone hydrochloride samples were placed in aluminum crucibles with perforated lids for mass determination. DSC (Model 1 Mettler Toledo) analysis was conducted at a heating rate of 10°C per minute, covering a temperature range from 30°C to 300°C, in a nitrogen atmosphere with a flow rate of 50 mL per minute. Data were processed using STARe software, licensed by Mettler Toledo.

### 2.5. Powder XRD Characterization

Buspirone hydrochloride samples were gently ground in a mortar with a pestle until a fine powder was obtained. XRD analysis was performed using a diffractometer (Miniflex 600 Rigaku Corporation) with Miniflex Guidance software (Rigaku), configured with the following parameters: 2*θ* angle range of 2–50°, step size of 0.020°, acquisition time of 40 s per step, DS slits set to 1/16°, scanning speed of 2.5° per minute, a 15 mm mask, sample‐to‐detector distance (AS) of 6.4 mm, copper X‐ray tube (Cu Kα1), D/Tex Ultra detector, and zero‐background sample holder (silicon).

### 2.6. Density and Flow Property Analysis

To analyze the bulk density of the buspirone hydrochloride samples, approximately 40 g of Samples IA or IIA were slowly transferred into a 250 mL graduated cylinder, and the resulting volume was recorded. For tapped density analysis, the sample was compacted using a tapped density tester, set to perform an initial 10 taps, followed by sequences of 500 and 1250 taps. Volume was recorded after each sequence. Tapping was repeated in increments of 1250 until the volume difference between consecutive measurements was less than 2 mL. Bulk density was calculated by dividing the initial sample mass (g) by the initial volume (mL) and expressed in g·mL^−1^. Tapped density was determined by dividing the initial sample mass (g) by the final compacted volume (mL), also expressed in g·mL^−1^.

The flow properties of buspirone hydrochloride samples were assessed through powder flowability, indicated by the Carr index, and compressibility, expressed by the Hausner ratio. Flow‐type classification followed the criteria established by Taylor and Aulton [13]. The Carr index (%) was calculated by subtracting the bulk density (g·mL^−1^) from the tapped density (g·mL^−1^), dividing by the tapped density (g·mL^−1^), and multiplying by 100 to yield a percentage. The Hausner ratio was calculated by dividing the tapped density (g·mL^−1^) by the bulk density (g·mL^−1^).

### 2.7. Solubility Analysis

A physicochemical analysis of the structural, thermal, and molecular properties of buspirone hydrochloride was conducted on control Samples IA and IIA, as well as stressed Samples IB, IIB, IC, and IIC, to identify mixtures of polymorphs and pure forms. Initial analyses suggested that control Sample IIA contained pure Form 2, while stressed Sample IIB contained pure Form 1; these findings were subsequently validated.

The solubility of pure Form 1 (Sample IIB) and Form 2 (Sample IIA) was assessed in various dissolution media designed to simulate the pH conditions of the human gastrointestinal tract, including hydrochloric acid (HCl) (0.1 mol·L^−1^, pH 1.2), sodium acetate buffer (SAB) (0.051 mol L^−1^, pH 4.5), and potassium phosphate buffer (KPB) (0.05 mol·L^−1^, pH 6.8). In triplicate, 50 mg of each sample (Form 1 or Form 2) was placed in amber glass vials with 20 mL of the respective dissolution medium, then incubated in an orbital shaker at 37°C and 150 rpm. After 24 h, a 5 mL aliquot was taken from each vial and filtered through a 0.45 μm polyvinylidene fluoride filter. Subsequently, 2 mL of the filtrate was transferred to a 10 mL volumetric flask and diluted to the 10 mL mark with 5 mL·mL^−1^ acetonitrile. This solution was used for quantification via high‐performance liquid chromatography (HPLC).

A 30 mg dose was selected to analyze the dose‐to‐solubility relationship, representing half of the maximum daily dose (60 mg) of buspirone hydrochloride recommended for the reference product (Ansitec; Libbs, Brazil). This dose aligns with the conservative recommendation to divide the daily dose into two doses. The dose‐to‐solubility ratio was calculated by dividing the highest concentration (mg) by the average concentration from triplicate measurements. Sample solubility was quantified using HPLC with reverse phase and UV/Vis detection (HPLC‐UV/Vis). The chromatographic system for solubility quantification was configured with parameters such as Mobile phase A: Mobile phase B (1:1), acetonitrile cleaning solution: purified water (1:9), acetonitrile injector solution: purified water (7:3), flow rate of 1.0 mL·min^−1^, injection volume of 1 μL, Macherey–Nagel Nucleosil​ C18 chromatographic column (150 mm × 4.6 mm × 5 μm), UV/Vis detector at 240 nm, and a run time of 8 min.

The solutions and mobile phases were prepared as follows: for Buffer solution A, 6.8 g of monobasic potassium phosphate and 0.93 g of sodium 1‐hexanesulfonate monohydrate were dissolved in 1000 mL of water, with the pH adjusted to 3.4 using 85% phosphoric acid. Mobile phase A was prepared by mixing 950 mL of Buffer solution A with 50 mL of acetonitrile, followed by degassing. Mobile phase B was prepared by dissolving 0.85 g of monobasic potassium phosphate and 0.88 g of sodium 1‐hexanesulfonate monohydrate in 250 mL of water, adjusting the pH to 2.2 with 85% phosphoric acid, then adding 750 mL of acetonitrile, followed by thorough homogenization and degassing. The 5 mL 100 mL^−1^ acetonitrile diluent was prepared by mixing 950 mL of water with 50 mL of acetonitrile, followed by thorough homogenization and degassing. The 10 mL 100 mL^−1^ acetonitrile solution was prepared by mixing 900 mL of water with 100 mL of acetonitrile, followed by thorough homogenization and degassing. Similarly, the 70 mL 100 mL^−1^ acetonitrile solution was prepared by combining 300 mL water with 700 mL acetonitrile, then homogenized and degassed to ensure consistency and removal of dissolved gases before use.

### 2.8. Statistical Analysis

All results were expressed as arithmetic mean values ± coefficient of variation when applicable. Differences between samples for each parameter were analyzed using the Student’s *t*‐test with a significance level of *α* = 0.01. Three samples were combined for all methodologies to create a representative composite, and all assays were conducted in triplicate.

## 3. Results

Two polymorphs of buspirone hydrochloride have been documented in the literature, referred to as Form P188 and Form P203 [8, 9] and as Form 1 and Form 2 [10, 11], underscoring a lack of standardized nomenclature. Although Sheikhzadeh et al. [10] cited Behme et al. [9], they did not apply the same terminology for these polymorphic forms. In our study, we adopted the terms Form 1 and Form 2 to establish a consistent nomenclature for these polymorphs.

**Figure 1:**
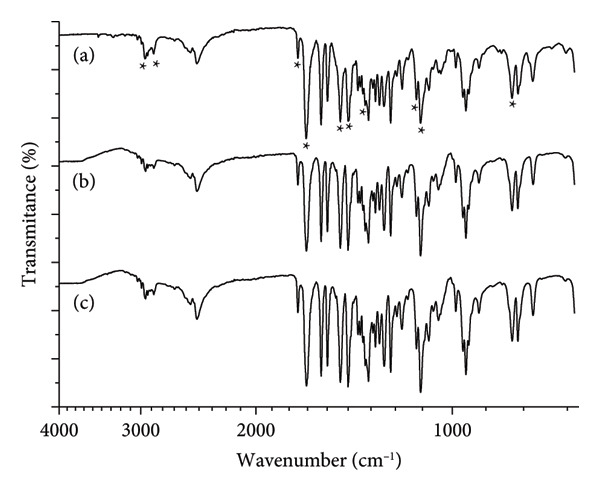
Fourier‐transform infrared (FTIR) spectrum (wavenumber versus transmittance in relative scale) of buspirone hydrochloride Sample I: (a) control Sample IA, (b) stored in open vial Sample IB, and (c) stored in closed vial Sample IC. Asterisks mark in the control the bands from left to right: 2953, 2865, 1723, 1673, 1484, 1444, 1359, 1270, 1119, and 809 cm^−1^.

Commercial buspirone hydrochloride from India, control Sample IA (Figure 1(a)), stressed Sample IB (Figure 1(b)), and stressed Sample IC (Figure 1(c)) were analyzed by FTIR. The analyses revealed characteristic molecular bands, including aliphatic C‐C bond stretching at 2953 and 2865 cm^−1^, amide C=O bond stretching at 1723 and 1673 cm^−1^, CH_2_ bond angular deformation at 1484 and 1444 cm^−1^, CH_3_ bond angular deformation at 1359 cm^−1^, aromatic C‐N bond stretching at 1270 cm^−1^, aliphatic C‐N bond stretching at 1119 cm^−1^, and the aromatic ring at 809 cm^−1^. These bands are marked with asterisks in Figure 1(a) (control) and are also present in the stressed Samples IB and IC (Figures 1(b) and 1(c)), though not marked in those figures to avoid visual clutter.

**Figure 2:**
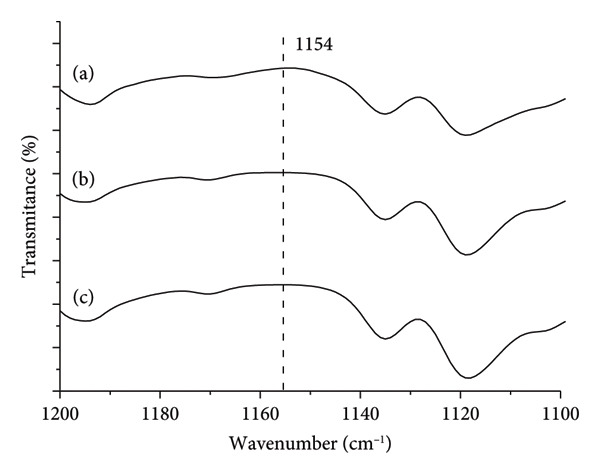
Expanded spectrum of the 1200–1100 cm^−1^ region (Figure 1) of Fourier‐transform infrared (FTIR) analysis (wavenumber versus transmittance in relative scale) of buspirone hydrochloride Sample I: (a) control Sample IA, (b) stored in open vial Sample IB, and (c) stored in closed vial Sample IC. The vertical line at 1154 cm^−1^ marks the place for the presence or absence of the vibrational band characteristic of the polymorph Form 2.

The absence of the vibrational band around 1154 cm^−1^, indicative of Form 2 according to Sheikhzadeh et al. [10], in the magnified region of Figure 1 (Figure 2; enlarged region 1200–1100 cm^−1^) suggests, by exclusion, that the samples consist of Form 1 of the active pharmaceutical ingredient.

**Figure 3:**
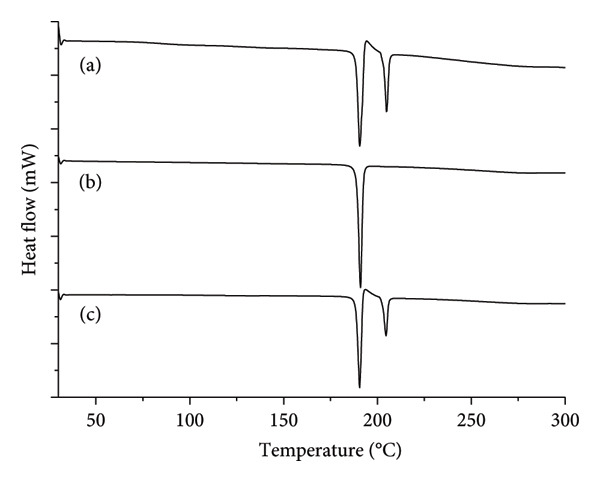
Differential scanning calorimetry (DSC) curves (temperature versus heat flow in relative scale) of buspirone hydrochloride Sample I: (a) control Sample IA, (b) stored in open vial Sample IB, and (c) stored in closed vial Sample IC.

The thermal analysis of buspirone hydrochloride from India by DSC revealed three distinct events: two endothermic and one exothermic, for both the control Sample IA (Figure 3(a)) and stressed Sample IC (Figure 3(c)). In contrast, the stressed Sample IB (Figure 3(b)) exhibited a single endothermic event. In the control Sample IA, the first endothermic event occurred at 188.22°C (ΔH fusion = −74.01 J·g^−1^), corresponding to the melting of Form 1. This was followed by an exothermic event at 193.17°C (ΔH recrystallization = −74.01 J·g^−1^), attributed to recrystallization, and a subsequent endothermic event at 202.89°C (ΔH fusion = −74.01 J·g^−1^), indicating the melting of Form 2. These results confirm the presence of both Form 1 and Form 2 in the control Sample IA.

Conversely, in stressed Sample IB (Figure 3(b)), the polymorphic mixture was fully converted to Form 1 after 48 days, as evidenced by a single endothermic event in the DSC curve at 188.62°C (ΔH fusion = −111.43 J·g^−1^), characteristic of Form 1. These results indicate that the sample is sensitive to changes in humidity and temperature, which facilitate the conversion from Form 2 to Form 1, suggesting that Form 1 is the more stable polymorph.

However, three distinct thermal events were observed for stressed Sample IC (Figure 3(c)), similar to control Sample IA. The first event is an endothermic peak at 188.28°C (ΔH fusion = −94.45 J·g^−1^), associated with the melting of Form 1, followed by an exothermic peak at 192.35°C (ΔH recrystallization = −13.49 J·g^−1^), related to the recrystallization process, and finally, another endothermic peak at 202.37°C (ΔH fusion = −37.95 J·g^−1^), indicative of the melting of Form 2. These results confirm the coexistence of Form 1 and Form 2 in Sample IC, with Form 2 present in a smaller proportion compared to Form 1 (Figure 3(c)). This suggests a predominant conversion of Form 2 to Form 1, with a residual fraction of Form 2 remaining, as indicated by the lower intensity and enthalpy values observed.

**Figure 4:**
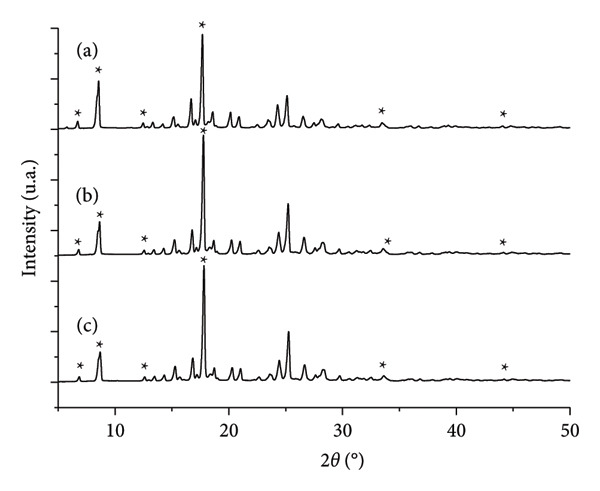
X‐Ray diffraction (XRD) patterns (°2*θ* versus intensity [units arbitrary] in relative scale) of powdered buspirone hydrochloride Sample I: (a) control Sample IA, (b) stored in open vial Sample IB, and (c) stored in closed vial Sample IC. Asterisks (^∗^) indicate characteristic peaks of polymorph Form 1.

In the XRD patterns, buspirone hydrochloride from India, control Sample IA (Figure 4(a)), stressed Sample IB (Figure 4(b)), and stressed Sample IC (Figure 4(c)) were analyzed. Characteristic diffraction peaks of Form 1 were identified and marked with an asterisk on each peak (Figure 4), located at 6.7, 8.5, 12.6, 17.6, 28.2, 33.6, and 44.8 (°2*θ*), as previously described by Sheikhzadeh et al. [10]. No diffraction peaks corresponding to Form 2 of the active pharmaceutical ingredient were detected.

**Figure 5:**
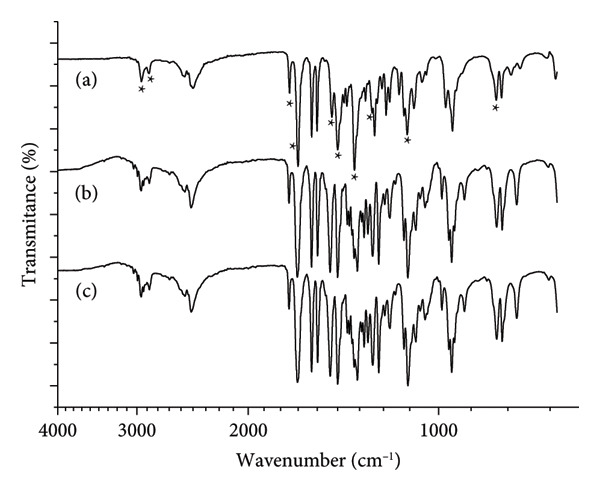
Fourier‐transform infrared (FTIR) spectrum (wavenumber versus transmittance in relative scale) of buspirone hydrochloride Sample II: (a) control Sample IIA, (b) stored in open vial Sample IIB, and (c) stored in closed vial Sample IIC. Asterisks (^∗^) mark in the control (a) the bands from left to right: 2953, 2865, 1723, 1673, 1484, 1444, 1359, 1270, 1119, and 809 cm^−1^.

Buspirone hydrochloride from Finland was analyzed by FTIR spectroscopy, including control Sample IIA (Figure 5(a)), stressed Sample IIB (Figure 5(b)), and stressed Sample IIC (Figure 5(c)). The analyses identified characteristic molecular bands, marked by asterisks in Figure 5(a) (control). Despite variations in band intensity observed, the spectral profiles of stressed Samples IIB (Figure 5(b)) and IIC (Figure 5(c)) remained similar.

**Figure 6:**
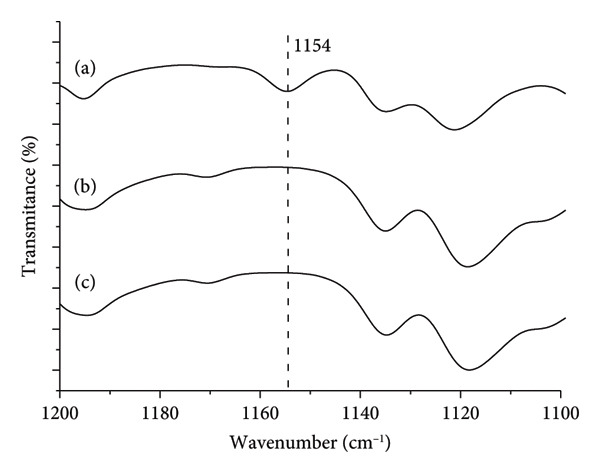
Expanded spectrum of the 1200–1100 cm^−1^ region (Figure 5) of Fourier‐transform infrared (FTIR) analysis (wavenumber versus transmittance in relative scale) of buspirone hydrochloride Sample II: (a) control Sample IIA, (b) stored in open vial Sample IIB, and (c) stored in closed vial Sample IIC. The vertical line at 1154 cm^−1^ marks the place for the presence or absence of the vibrational band characteristic of polymorph Form 2.

The vibrational band around 1154 cm^−1^, characteristic of Form 2 as reported by Sheikhzadeh et al. [10], was distinctly observed in the magnified region of Figure 6 in control Sample IIA (Figure 6(a)). However, this characteristic band of Form 2 was absent in the spectra of stressed Samples IIB (Figure 6(b)) and IIC (Figure 6(c)), suggesting a conversion from Form 2 to Form 1 in the active pharmaceutical ingredient after 48 days of exposure.

**Figure 7:**
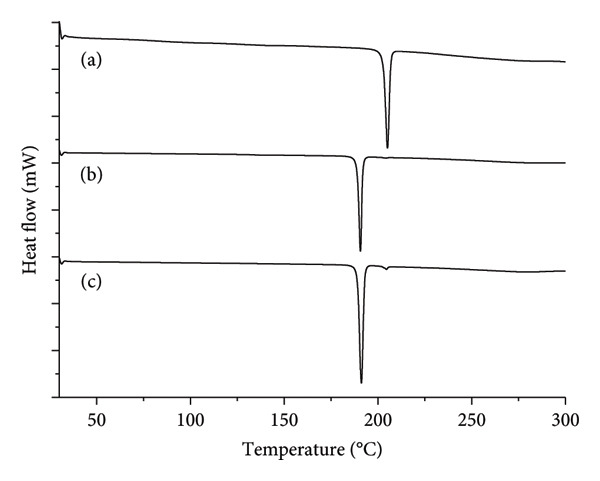
Differential scanning calorimetry (DSC) curves (temperature versus heat flow in relative scale) of buspirone hydrochloride Sample II: (a) control Sample IIA, (b) stored in open vial Sample II, and (c) stored in closed vial Sample IIC.

The thermal analysis of buspirone hydrochloride from Finland, conducted by DSC, revealed two distinct patterns: a single endothermic event in both control Sample IIA (Figure 7(a)) and stressed Sample IIB (Figure 7(b)) and two endothermic events in stressed Sample IIC (Figure 7(c)). In control Sample IIA, the endothermic event occurred at 202.71°C (ΔH fusion = −97.04 J·g^−1^), indicating the melting of Form 2. In contrast, stressed Sample IIB showed an endothermic event at 188.58°C (ΔH fusion = −115.13 J·g^−1^), associated with the melting of Form 1. Stressed Sample IIC presents a first endothermic event at 188.72°C (ΔH fusion = −115.68 J·g^−1^), indicative of Form 1 melting, followed by a subtler endothermic event at 202.01°C (ΔH fusion = −2.54 J·g^−1^), marking the melting of Form 2, with no recrystallization event detected. These results suggest that, while a minor residual fraction of Form 2 persisted in stressed Sample IIC, the polymorphic conversion was predominantly from Form 2 to Form 1.

**Figure 8:**
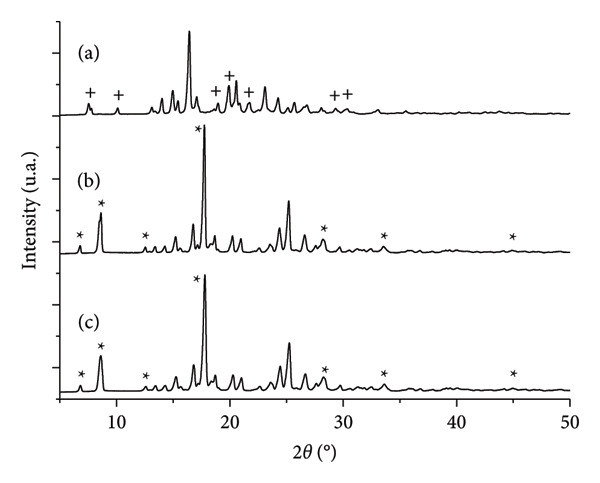
X‐Ray diffraction (XRD) patterns (°2*θ* versus Intensity [units arbitrary] in relative scale) of powdered buspirone hydrochloride Sample II: (a) control Sample IIA, (b) stored in open vial Sample IIB, and (c) stored in closed vial Sample IIC. Plus signs (+) indicate characteristic peaks of Form 2, while the asterisks (^∗^) indicate characteristic peaks of Form 1 of the polymorph.

In the XRD patterns of buspirone hydrochloride from Finland, control Sample IIA (Figure 8(a)) and stressed Samples IIB (Figure 8(b)) and IIC (Figure 8(c)) were examined. Diffraction peaks representative of Form 2 were identified in control Sample IIA (Figure 8(a)) and were marked with plus signs at 7.4, 10.0, 18.9, 20.0, 21.7, 29.3, and 30.3 (°2*θ*), consistent with Form 2 characteristic peaks reported by Sheikhzadeh et al. [10]. In contrast, diffraction peaks characteristic of Form 1 were identified in stressed Samples IIB (Figure 8(b)) and IIC (Figure 8(c)), marked with asterisks at 6.7, 8.5, 12.6, 17.6, 28.2, 33.6, and 44.8 (°2*θ*), aligning with descriptions of Form 1 from Sheikhzadeh et al. [10]. No diffraction peaks corresponding to Form 2 were detected in stressed Samples IIB and IIC.

Thermal analysis by DSC proved to be the most accurate method for identifying the polymorphic forms of buspirone hydrochloride, as demonstrated by the results obtained from Sample I (India, Figure 3) and Sample II (Finland, Figure 7). In contrast, FTIR spectroscopy (Figures 1 and 5) and XRD patterns (Figures 4 and 8) were not effective in detecting mixtures of Forms 1 and 2, especially in stressed Sample IB (Figures 1 and 4). Therefore, thermal analysis by DSC is the most reliable detection method for samples containing polymorphic mixtures.

Density (Table 1) and flow (Table 2) analyses of buspirone hydrochloride samples revealed an unsatisfactory flow, classified as “very, very poor.” This classification is evident from the difference between bulk and tapped densities, resulting in high Carr index and Hausner ratio values (Table 2). Control Sample IA, which contained a mixture of Forms 1 and 2, showed slightly better Carr index and Hausner ratio values than control Sample IIA, which was predominantly composed of Form 2. Nonetheless, both samples remained within the “very, very poor” flow classification (Table 2). Consequently, for formulation development, incorporating lubricants and/or applying wet granulation techniques is recommended to reduce variability in the production process. These measures are essential, as the density and flow properties indicate that the raw material is unsuitable for direct compression processes.

**Table 1:** Analysis of bulk density and tapped density of buspirone hydrochloride: control Sample IA from India and Sample IIA from Finland.

Parameter	Sample IA	Sample IIA
Mass (g)	40.9273	40.4753
V0 (mL)	200	236
V10 (mL)	175	218
V500 (mL)	106	112
V1250 (mL)	103	109
V1250 (mL) add.	103	108
Bulk density (g mL^−1^)	0.2046	0.1715
Tapped density (g mL^−1^)	0.3974	0.3748

*Note:* V0 = initial bulk volume; V10 = volume after 10 taps; V500 = volume after 500 taps; V1250 = volume after 1250 taps; V1250 add. = volume after an additional 1250 taps. Bulk density is calculated by dividing the sample mass by its bulk volume (V0). Tapped density is calculated by dividing the sample mass by its tapped volume (V1250 add.).

**Table 2:** Classification of powder flow properties based on compressibility (Carr index) and particle friction (Hausner ratio) of buspirone hydrochloride: control Sample IA from India and Sample IIA from Finland.

Parameter	Sample IA	Sample IIA
Carr index (%)	48.5153	54.2423
Flow type^∗^	Very, very poor	Very, very poor
Hausner ratio	1.9423	2.1854
Flow type^∗^	Very, very poor	Very, very poor

^∗^Flow type = classification of powder flow properties according to Carr index or Hausner ratio, according to Taylor and Aulton [13]. The Carr index is obtained by subtracting bulk density from tapped density, dividing by tapped density, multiplying by 100, and expressing the result as a percentage. Hausner ratio is determined by dividing tapped density by bulk density.

Solubility analysis was conducted on buspirone hydrochloride control Sample IIA, representing the pure Form 2 polymorph, and stressed Sample IIB, representing the pure Form 1. The solubility and dose‐to‐solubility ratio of pure Form 1 and Form 2 were evaluated after 24 h (Table 3).

**Table 3:** Solubility analysis of buspirone hydrochloride control Sample IIA (pure Form 1) and stressed Sample IIB (pure Form 2), conducted in dissolution media of hydrochloric acid (HCl; pH 1.2), sodium acetate buffer (SAB; pH 4.5), and potassium phosphate buffer (KPB; pH 6.8), along with an analysis of dose‐to‐solubility ratio (D/S).

Polymorph	HCl (pH 1.2) solubility^∗^ (mg mL^−1^)	D/S ratio^∗∗^	SAB (pH 4.5) solubility^∗^ (mg mL^−1^)	D/S ratio^∗∗^	KPB (pH 6.8) solubility^∗^ (mg mL^−1^)	D/S ratio^∗∗^
Form 1	2.55 ± 0.99	11.8	2.55 ± 0.17	11.8	1.25 ± 0.41	24.0
Form 2	2.57 ± 0.37	11.7	2.58 ± 1.10	11.6	1.26 ± 0.60	23.8

*Note:* Values expressed as arithmetic mean ± coefficient of variation (*n* = 3).

^∗^Solubility values in the same column do not differ significantly according to Student’s *t*‐test (*p* ≤ 0.01).

^∗∗^The highest concentration of 30.0 mg buspirone hydrochloride was used as a reference for the Biopharmaceutical Classification System (BCS) [14].

Pure Form 1 and Form 2 of buspirone hydrochloride showed equivalent solubility profiles (Table 3), demonstrating that polymorphic differences in crystalline structure and molecular interactions do not affect solubility. A 30.0 mg dose of each polymorph, representing the maximum recommended intake, fully dissolved in less than 250 mL of dissolution media over a physiological pH range of 1.2–6.8 at 37°C, resulting in an optimal dose‐to‐solubility ratio (Table 3). This bioavailability assessment suggests that both Form 1 and Form 2 can be equally well absorbed in gastric and intestinal environments, making them suitable for immediate‐release formulations.

The coefficient of variation for solubility analysis remained below 5% (Table 3), complying with the standards recommended by the Brazilian Pharmacopoeia for pharmaceutical quality [15]. Both polymorphs exhibited decreased solubility as pH increased, with the lowest solubility observed in KPB at pH 6.8 (Table 3). This pH‐dependent solubility profile indicates that both Form 1 and Form 2 of buspirone hydrochloride are more soluble under acidic conditions and less soluble in near‐neutral environments. Nonetheless, this variation did not affect the overall dissolution profile, as solubility remained sufficiently high across the tested pH range (Table 3).

Given the high solubility of both Form 1 and Form 2 of buspirone hydrochloride, they can be tentatively classified as either Class I or Class III under the BCS [14]. However, the absence of permeability data precludes a definitive classification, especially considering the polymorphic nature of the compound. These findings demonstrate that solubility‐related polymorph control is unnecessary, as both forms exhibit similar solubility characteristics. Consequently, from a solubility standpoint, the polymorphism of buspirone hydrochloride is unlikely to impact its pharmaceutical formulations’ quality, safety, or efficacy.

## 4. Discussion

Buspirone, an anxiolytic drug belonging to the azapirone class [7], is commercially available as buspirone hydrochloride; however, it does not have a standardized polymorphic form. Polymorphs with higher melting points exhibit greater stability [16, 17]. However, for buspirone hydrochloride, the lower‐melting Form 1 (P188) is more stable, which led Simms [8] to recommend Form 1 for medications due to its resistance to conversion into Form 2. A similar situation was reported with the polymorphs of famotidine, where the lower‐melting form demonstrated greater stability [18]. Nonetheless, Sheikzadeh et al. [10] described buspirone hydrochloride polymorphs as enantiotropic, suggesting that this distinction is less critical, as the forms can reversibly transform between each other. In our study, buspirone hydrochloride has been sold as a mixture of polymorphs (control Sample IA from India) or pure Form 2 (control Sample IIA from Finland). Moreover, under environmental conditions such as high temperature and humidity, Form 2 or mixtures of Form 1 and Form 2 tend to convert to the more stable Form 1. However, it should be acknowledged that the enantiotropic behavior of this compound could facilitate continuous interconversion between polymorphic forms, although Form 1, being more stable, is generally favored. Consequently, Sample IIA from Finland, comprising pure Form 2, was probably subjected to strict humidity and temperature control during production, storage, and transportation. Such meticulous handling was essential to preserve its purity and stability, especially during intercontinental shipment, to prevent its conversion to Form 1.

The polymorphic forms of buspirone hydrochloride raise product consistency and therapeutic effectiveness concerns. Specifically, the potential conversion of Form 2 to the more stable Form 1 requires strict control of storage and processing conditions, particularly temperature and humidity, as these factors accelerate the transformation. In our study, control Sample IA, a mixture of Form 1 and Form 2, maintained a similar composition when stored in a closed vial at 75% relative humidity and 50°C for 48 days, resulting in Sample IIC. In contrast, Sample IIB, stored under identical conditions but in an open vial, was fully converted to Form 1. These findings confirm that airtight packaging could mitigate polymorphic conversion, which is essential in ensuring medication consistency. Therefore, controlled storage and packaging are fundamental for maintaining the stability of buspirone hydrochloride’s polymorphic forms.

XRD is widely regarded as the gold standard for studying compound polymorphism [19]. Lou and Zuo [20] successfully employed XRD to characterize and quantify losartan potassium Forms I and III. However, in our study, XRD was ineffective for analyzing buspirone hydrochloride polymorphism, as it was challenging to distinguish between the two mixed polymorphs. This limitation is likely due to the similarity in the diffraction patterns of Form 1 and Form 2, with Form 2 displaying more distinct peaks at lower 2‐theta angles and lower intensities. In contrast, higher‐intensity peaks are typically more precise and suitable for identifying and quantifying mixtures. More precise and detailed analytical techniques are required to address these challenges, including quantitative methods and refinements based on crystallographic data. High‐resolution software can help minimize peak overlap and mask low‐intensity peaks from minor phases. In addition, slower scan rates can reduce noise and improve peak definition, particularly in overlapping regions and areas of low intensity [20, 21].

FTIR analysis, which relies on the specific vibration frequencies of chemical bonds, is a valuable tool for studying molecules’ vibrational characteristics and crystalline properties. Frelek et al. [22] successfully used this technique to characterize Forms I, II, and III of finasteride. In our study of buspirone hydrochloride, however, clear identification of polymorphs was challenging, except for the 1154 cm^−1^ vibrational band, which serves as a reference [10]. This limitation likely stemmed from many similar vibrational bands across polymorphic forms and intermolecular interactions among functional groups, which did not present significant distinctions. Brittain [23] reported that for spectroscopy to produce meaningful results, vibrational modes must be sufficiently perturbed by crystallographic differences. Donahue et al. [24] reported that FTIR spectra for various polymorphic forms in complex molecular structures often display similar patterns, complicating differentiation. Due to molecular interactions, slight band intensities and frequency variations are typically insufficient for clear identification.

Furthermore, significant band overlap can obscure characteristic vibrational bands essential for polymorph identification and quantification, limiting the sensitivity and specificity of FTIR as a sole analytical tool. Gangrade et al. [25] suggest that combining FTIR with other techniques, such as XRD and DSC, enhances the precision of polymorphic identification. In our study, while FTIR provided valuable insights, DSC was essential for accurately identifying polymorphic mixtures of buspirone hydrochloride.

DSC measures the heat flow required to maintain a constant temperature between a sample and a reference [26]. The area under a DSC peak corresponds to the heat absorbed or released during phase transitions, such as melting or crystallization [1, 26]. This technique has effectively characterized polymorphs in compounds such as bisoprolol fumarate [27]. In our study, DSC surpasses FTIR and XRD in precision, especially in identifying polymorphs within buspirone hydrochloride mixtures, providing a clear visual differentiation of specific forms. This advantage likely arises from the marked differences in melting points and enthalpies between the two polymorphs and DSC’s high sensitivity to impurities. Bruni et al. [28] also found that DSC outperformed FTIR and XRD in detecting polymorph mixtures in dexketoprofen trometamol and identified impurities as low as 0.3%. In addition, studies on nateglinide [28], eletriptan hydrobromide [29], and dexketoprofen trometamol [28], employing DSC, FTIR, and XRD, consistently showed DSC’s superior selectivity and effectiveness in identifying and quantifying polymorphic mixtures. These findings underscore DSC as a highly effective and reliable technique for polymorphic analysis, particularly in polymorphic mixtures of buspirone hydrochloride.

Buspirone hydrochloride, in polymorphic forms and mixtures, was classified in our study as having “very, very poor” flow properties, presenting a substantial challenge for efficient pharmaceutical manufacturing. Achieving consistent mixing and accurate dosing in solid drug formulations requires improving these properties beforehand, as inadequate flow can lead to significant inconsistencies. One approach to improve flow includes incorporating lubricating excipients such as magnesium stearate, sodium stearyl fumarate, sodium lauryl sulfate, stearic acid, and poloxamers, as well as glidants such as colloidal silica, which aid powder handling [30, 31]. Magnesium stearate remains the most recommended excipient due to its high lubrication efficiency [30, 32]. However, alternatives are also considered since magnesium stearate exhibits polymorphic variation, differing in hydration level, particle size, and phase composition (crystalline and amorphous), which can affect its lubrication performance [33].

In addition, it has been associated with tablet friability issues and delayed dissolution [30, 31]. Beyond excipient selection, modifying granulation and compression techniques provides further strategies to address poor flow properties. Wet granulation, for example, is widely used to improve flow and compressibility [31, 34], while co‐crystal formation can enhance powder flow characteristics [35]. These approaches improve the manufacturing efficiency of this pharmaceutical product by addressing flow properties, resulting in enhanced quality, efficacy, and safety.

Although static flowability measurements such as Carr’s index and Hausner ratio are widely used for preliminary assessment of powder behavior, they present limitations when predicting performance under actual processing conditions. These static methods do not fully capture the complex, dynamic interactions that occur during manufacturing steps such as blending, feeding, granulation, or compression. In our study, only static flowability tests were employed due to methodological scope and instrumentation constraints. However, we recognize the importance of incorporating dynamic flow characterization techniques, including powder rheometry and shear cell testing, in future studies to more accurately simulate and understand the behavior of polymorphic forms during solid dosage manufacturing. Such approaches offer enhanced reproducibility and industrial relevance, as emphasized in recent reviews by Shah et al. [36] and Moravkar et al. [37], which highlights the need for integrating both traditional and advanced methods for comprehensive powder flow evaluation.

Buspirone hydrochloride exhibits pH‐dependent solubility in polymorphic Form 1 and Form 2, with lower solubility in KPB at pH 6.8. This reduced solubility is attributed to limited ionization, as the drug predominantly exists in a less soluble ionized form, and the formation of soluble salts [38]. At pH 6.8, this low solubility can also affect chemical stability, leading to degradation or precipitation, further reducing solubility [39]. Consequently, this reduced solubility at high pH levels, such as in the intestinal environment, can hinder drug absorption and thus lower bioavailability [38]. Addressing this challenge requires strategies to improve solubility at pH 6.8, including solubility enhancers or modifications to the pharmaceutical formulation, regardless of the polymorph form.

In our study, Form 1 and Form 2 of buspirone hydrochloride exhibit similar solubility characteristics. However, Sheikhzadeh et al. [11] reported that Form 2 has higher solubility in water and isopropanol than Form 1. It is important to note that this assessment by Sheikhzadeh et al. [11] was conducted under conditions that do not replicate physiological conditions. Consequently, although the differences in solubility observed in specific solvents are valid, the high solubility of both polymorphic forms (Form 1 and Form 2) of buspirone hydrochloride remains consistently supported within the BCS.

Buspirone hydrochloride exhibits high solubility in both of its known polymorphic forms (Form 1 and Form 2), supporting its preliminary classification within BCS Class I. BCS Class I compounds are characterized by high solubility and high permeability, whereas Class III compounds share high solubility but exhibit low permeability [14]. Regulatory agencies, including the U.S. FDA, have recognized buspirone hydrochloride as eligible for a BCS Class I–based biowaiver [40]. Similarly, the Swedish Medical Products Agency (MPA) endorsed this classification in its Public Assessment Report, granting a BCS‐based biowaiver for a generic formulation. This decision was based on documented evidence of high solubility, high permeability, and the absence of a narrow therapeutic index [12], justifying the waiver of in vivo bioequivalence studies. Despite these regulatory endorsements, none of the cited studies specify which polymorphic form of buspirone hydrochloride was assessed in solubility and permeability evaluations. Although solubility has been shown to remain unaffected by polymorphism in this case (our study results), the impact of polymorphic variation on permeability remains uncertain. Considering the reversible interconversion between Form 1 and Form 2 and the current lack of permeability data specific to each form, it is plausible that one polymorph, or a specific mixture, possesses high permeability, whereas the other might not exhibit the same level of permeability. Thus, although buspirone hydrochloride is generally classified as a BCS Class I compound, a definitive classification for each polymorphic form has yet to be established. Depending on their unique permeability characteristics, either form could conceivably fall under BCS Class I or Class III. Therefore, further studies assessing the permeability of Form 1 and Form 2 independently are warranted to refine their biopharmaceutical classification and confirm their respective capacities for gastrointestinal absorption and oral bioavailability.

Understanding the polymorphic properties of buspirone hydrochloride is essential for optimizing drug manufacturing, transport, and storage. Although both polymorphs demonstrate high solubility, their permeability remains uncertain. In addition, both forms exhibit poor flow properties and low density, presenting challenges in handling, blending, and compressing the material into tablets or capsules. Consequently, further research is necessary to enhance the manufacturing processes for buspirone hydrochloride.

## 5. Conclusion

Buspirone hydrochloride exists in two polymorphic forms, Form 1 and Form 2, which readily interconvert, especially under varying temperatures and humidity, with Form 1 as the more stable form. Commercially, Sample IA from India contains a mixture of Forms 1 and 2, whereas Sample IIA from Finland consists solely of Form 2. The conversion of buspirone hydrochloride from Form 2 to Form 1 accelerates with increased temperature and humidity, but complete conversion occurs at different times depending on the sample’s origin. DSC thermal analysis effectively detects both polymorphic forms in buspirone hydrochloride, particularly in mixtures, whereas XRD and FTIR are less effective for this purpose. The flow properties and low density of both Forms 1 and 2 of buspirone hydrochloride are classified as “very, very poor,” posing significant challenges for efficient pharmaceutical manufacturing. Both polymorphic forms exhibit pH‐dependent solubility, which is more soluble under acidic conditions and less soluble in near‐neutral environments. The solubility of Forms 1 and 2 of buspirone hydrochloride is high and equivalent, classifying them within BCS Class I or Class III. However, the permeability profiles of the two polymorphic forms remain unclear and warrant further investigation. These findings advance the understanding of buspirone hydrochloride’s polymorphic properties, informing the development of more effective formulations. Future studies should investigate strategies to improve these polymorphs’ flow properties and permeability and explore molecular interactions that could impact pharmaceutical efficacy and manufacturing processes.

## Disclosure

All authors have read and approved the final version of the manuscript.

## Conflicts of Interest

Jéssika Adriane Janning and Márcia Nunes da Silva, both employed at Prati‐Donaduzzi Pharmaceutical Company, disclose a potential conflict of interest due to their employment during Janning’s Master′s degree research at the Federal University of Technology of Paraná (UTFPR). The other authors declare no conflicts of interest.

## Author Contributions

Jéssika Adriane Janning was responsible for conceptualization, data curation, formal analysis, investigation, methodology, visualization, and writing–original draft. Victória Guimarães Santiago contributed to data curation, formal analysis, investigation, and writing–original draft. Ederlan de Souza Ferreira, Carolina Oliveira de Souza, Márcia Nunes da Silva, Filipa Sofia Reis, and Nelson Barros Colauto were involved in formal analysis, methodology, validation, and writing–review and editing. Renato Eising contributed to conceptualization, funding acquisition, methodology, project administration, supervision, and writing–review and editing. All authors assure that the research was conducted with complete objectivity and impartiality.

## Funding

The authors would like to express their gratitude to the following institutions for their support: The Federal University of Technology‐Paraná; the Scientific and Technological Park of Biosciences (Biopark); the Coordination for the Improvement of Higher Education Personnel, Brazil (CAPES)‐financial code 001‐; the Araucária Foundation; and the National Council for Scientific and Technological Development (CNPq), Brazil. The authors also acknowledge the Foundation for Science and Technology (FCT), Portugal, for national funding provided through the individual scientific employment program contract (2021.03728.CEECIND).

## Data Availability

The data that support the findings of this study are available from the corresponding author upon reasonable request.

## References

[1] Craig D. Q. M. , Hilfiker R. , Characterization of Polymorphic Systems Using Thermal Analysis, Polymorphism, 2006, Wiley‐VCH Verlag Gmbh & Co. Kgaa, Weinheim, 43–79, 10.1002/3527607889.ch3, 2-s2.0-33746645606.

[2] Santos O. M. M. , Reis M. E. D. , Jacon J. T. , Lino M. E. d.S. , Simões J. S. , and Doriguetto A. C. , Polymorphism: An Evaluation of the Potential Risk to the Quality of Drug Products From the Farmácia Popular Rede Própria, Brazilian Journal of Pharmaceutical Sciences. (2014) 50, no. 1, 1–11, 10.1590/S1984-82502011000100002, 2-s2.0-84900390500.

[3] Aguiar A. J. , Krc J.Jr., Kinkel A. W. , and Samyn J. C. , Effect of Polymorphism on the Absorption of Chloramphenicol From Chloramphenicol Palmitate, Journal of Pharmaceutical Sciences. (1967) 56, no. 7, 847–853, 10.1002/jps.2600560712, 2-s2.0-0014104395. 6034828

[4] Agency E. M. , E.M.A. Cpmp/ICH/367/96, Q6A Specifications: Test Procedures and Acceptance Criteria for New Drug Substances and New Drug Products: Chemical Substances: Scientific Guideline, Emea, 2000, London. 12356095

[5] AnviSA A. N. d.V. S.-. , AnviSA, ResoLUÇÃO DE DireTORIA ColeGIADA: RDC N° 361, DE 27 DE MarçO DE 2020, Diário Oficial da União - DOU, 2020, Brasília.

[6] FDA , Guidance for Industry: Andas: Pharmaceutical Solid Polymorphism Chemistry, Manufacturing, and Controls Information in. (2007) U.S.D.o.H.a.H. Services, Rockville, MD.

[7] Du Y. , Li Q. , Dou Y. et al., Side Effects and Cognitive Benefits of Buspirone: A Systematic Review and Meta-Analysis, Heliyon. (2024) 10, no. 7, 10.1016/j.heliyon.2024.e28918. PMC11004816 38601569

[8] Simms J. C. , Pharmaceutical Useful Polymorphic Modification of Buspirone (Bristol Myers Co/Bristol Myers Squibb Co, United States), 1991, https://patents.google.com/patent/US5015646A/en.

[9] Behme R. J. , Kensler T. T. , and Mikolasek D. G. , Process for Buspirone Hydrochloride Polymorphic Crystalline Form Conversion, 1989, Bristol-Myers Company, United States, https://patents.google.com/patent/US4810789A/en.

[10] Sheikhzadeh M. , Rohani S. , Jutan A. , Manifar T. , Murthy K. , and Horne S. , Solid-State Characterization of Buspirone Hydrochloride Polymorphs, Pharmaceutical Research. (2006) 23, no. 5, 1043–1050, 10.1007/s11095-006-9779-6, 2-s2.0-33646876184. 16715396

[11] Sheikhzadeh M. , Rohani S. , Jutan A. , and Manifar T. , Quantitative and Molecular Analysis of Buspirone Hydrochloride Polymorphs, Journal of Pharmaceutical Sciences. (2007) 96, no. 3, 569–583, 10.1002/jps.20723, 2-s2.0-33947526889. 17094124

[12] Swedish Medical Products Agency , Public Assessment Report: Buripal (Buspirone, Buspirone Hydrochloride) 5 Mg, 10 Mg Tablets, 2024, Swedish MPA, https://www.lakemedelsverket.se.

[13] Taylor K. M. G. and Aulton M. E. , Aulton’s Pharmaceutics: The Design and Manufacture of Medicines, 2021, 6th edition, Elsevier, https://shop.elsevier.com/books/aultons-pharmaceutics/taylor/978-0-7020-8154-5.

[14] Amidon G. L. , Lennernäs H. , Shah V. P. , and Crison J. R. , A Theoretical Basis for a Biopharmaceutic Drug Classification: The Correlation of *in Vitro* Drug Product Dissolution and *in Vivo* Bioavailability, Pharmaceutical Research. (1995) 12, no. 3, 413–420, 10.1023/a:1016212804288, 2-s2.0-0028948839. 7617530

[15] Brazil, Brazilian Pharmacopoeia, 2019, 6th edition, AnviSA, https://www.gov.br/anvisa/pt-br/english/pharmacopeia.

[16] Braga D. , Casali L. , and Grepioni F. , The Relevance of Crystal Forms in the Pharmaceutical Field: Sword of Damocles or Innovation Tools?, International Journal of Molecular Sciences. (2022) 23, no. 16, 10.3390/ijms23169013. PMC9408954 36012275

[17] Moondra S. , Maheshwari R. , Taneja N. , Tekade M. , and Tekadle R. K. , Tekade R. K. , Chapter 6: Bulk Level Properties and Its Role in Formulation Development and Processing, Dosage Form Design Parameters, 2018, Academic Press, 221–256, 10.1016/B978-0-12-814421-3.00006-3.

[18] Soto R. and Svärd M. , Solubility and Thermodynamic Analysis of Famotidine Polymorphs in Pure Solvents, International Journal of Pharmaceutics. (2021) 607, 10.1016/j.ijpharm.2021.121031. 34419593

[19] Singh P. , Sharma S. , Sharma P. K. , and Alam A. , Drug Polymorphism: An Important Pre-Formulation Tool in the Formulation Development of a Dosage Form, Current Physical Chemistry. (2024) 14, no. 1, 2–19, 10.2174/1877946813666230822113606.

[20] Lou Y. and Zuo L. , Quantification of Losartan Potassium Polymorphs Using Powder X-Ray Diffraction, Journal of Aoac International. (2020) 104, no. 3, 579–584, 10.1093/jaoacint/qsaa166. 33337486

[21] Qiu J.-B. , Li G. , Sheng Y. , and Zhu M.-R. , Quantification of Febuxostat Polymorphs Using Powder X-Ray Diffraction Technique, Journal of Pharmaceutical and Biomedical Analysis. (2015) 107, 298–303, 10.1016/j.jpba.2015.01.005, 2-s2.0-84921696265. 25636167

[22] Frelek J. , Górecki M. , Dziedzic A. et al., Comprehensive Spectroscopic Characterization of Finasteride Polymorphic Forms. Does the Form X Exist?, Journal of Pharmaceutical Sciences. (2015) 104, no. 5, 1650–1657, 10.1002/jps.24369, 2-s2.0-84926683010. 25648836

[23] Brittain H. G. , Characterization of Pharmaceutical Compounds in the Solid State, Separation Science and Technology. (2011) 10, 11–58, 10.1016/B978-0-12-375680-0.00002-4, 2-s2.0-78649937196.

[24] Donahue M. , Botonjic-Sehic E. , Wells D. , and Brown C. W. , Understanding Infrared and Raman Spectra of Pharmaceutical Polymorphs, American Pharmaceutical Review. (2011) 14, no. 2, https://www.americanpharmaceuticalreview.com/Featured-Articles/37183-Understanding-Infrared-and-Raman-Spectra-of-Pharmaceutical-Polymorphs/.

[25] Gangrade D. , Sharma A. , and Bakshi S. , Determination of Polymorphic Forms of Finasteride by Xrpd, FT-IR, and DSC Techniques, World Journal of Pharmaceutical Sciences. (2015) 3, 1892–1898, https://wjpsonline.com/index.php/wjps/article/view/polymorphic-forms-finasteride-xrpd-ftir-dsc-techniques.

[26] Brittain H. G. , Brittain H. G. , Methods for the Characterization of Polymorphs and Solvates, Polymorphism in Pharmaceutical Solids, 1999, M. Dekker, New York.

[27] Detrich Á. , Dömötör K. J. , Katona M. T. , Markovits I. , and Vargáné Láng J. , Polymorphic Forms of Bisoprolol Fumarate, Journal of Thermal Analysis and Calorimetry. (2019) 135, no. 6, 3043–3055, 10.1007/s10973-018-7553-8, 2-s2.0-85050336545.

[28] Bruni G. , Capsoni D. , Milanese C. , and Cardini A. , Polymorphic Quantification of Dexketoprofen Trometamol by Differential Scanning Calorimetry, Journal of Thermal Analysis and Calorimetry. (2023) 148, no. 5, 1949–1958, 10.1007/s10973-022-11870-Y.

[29] Kommavarapu P. , Maruthapillai A. , and Palanisamy K. , Identification and Quantitative Determination of Eletriptan Hydrobromide Polymorphs: Thermal, Diffractometric and Spectrometric Studies, Journal of Taibah University for Science. (2015) 9, no. 4, 586–593, 10.1016/j.jtusci.2015.03.011.

[30] Dun J. , Osei-Yeboah F. , Boulas P. , Lin Y. , and Sun C. C. , A Systematic Evaluation of Dual Functionality of Sodium Lauryl Sulfate as a Tablet Lubricant and Wetting Enhancer, International Journal of Pharmaceutics. (2018) 552, no. 1-2, 139–147, 10.1016/j.ijpharm.2018.09.056, 2-s2.0-85054191813. 30261213

[31] Wang J. , Wen H. , and Desai D. , Lubrication in Tablet Formulations, European Journal of Pharmaceutics and Biopharmaceutics. (2010) 75, no. 1, 1–15, 10.1016/j.ejpb.2010.01.007, 2-s2.0-77951652900. 20096779

[32] Zuurman K. , Van der Voort Maarschalk K. , and Bolhuis G. K. , Effect of Magnesium Stearate on Bonding and Porosity Expansion of Tablets Produced From Materials With Different Consolidation Properties, International Journal of Pharmaceutics. (1999) 179, no. 1, 107–115, 10.1016/s0378-5173(98)00389-5, 2-s2.0-0032966271. 10053207

[33] Zarmpi P. , Flanagan T. , Meehan E. , Mann J. , and Fotaki N. , Impact of Magnesium Stearate Presence and Variability on Drug Apparent Solubility Based on Drug Physicochemical Properties, The Aaps Journal. (2020) 22, no. 4, 10.1208/s12248-020-00449-W. PMC7242257 32440810

[34] Moravkar K. K. , Shah D. S. , Magar A. G. et al., Assessment of Pharmaceutical Powders Flowability and Comparative Evaluation of Lubricants on Development of Gastro Retentive Tablets: An Application of Powder Flow Tester, Journal of Drug Delivery Science and Technology. (2022) 71, 10.1016/j.jddst.2022.103265.

[35] Nijhawan M. , Godugu M. , Saxena T. , Farheen T. , and Dwivedi K. , Pharmaceutical Co-Crystals of Posaconazole for Improvement of Physicochemical Properties, Brazilian Journal of Pharmaceutical Sciences. (2022) 58, 10.1590/s2175-97902022e191024.

[36] Shah D. S. , Moravkar K. K. , Jha D. K. , Lonkar V. , Amin P. D. , and Chalikwar S. S. , A Concise Summary of Powder Processing Methodologies for Flow Enhancement, Heliyon. (2023) 9, no. 6, 10.1016/j.heliyon.2023.e16498. PMC10245010 37292344

[37] Moravkar K. K. , Korde S. D. , Bhairav B. A. , Shinde S. B. , Kakulade S. V. , and Chalikwar S. S. , Traditional and Advanced Flow Characterization Techniques: A Platform Review for Development of Solid Dosage Form, Indian Journal of Pharmaceutical Sciences. (2020) 82, no. 6, 945–957, 10.36468/pharmaceutical-Sciences.726.

[38] An Q. , Xing C. , Wang Z. et al., Metformin-Mediated Improvement in Solubility, Stability, and Permeability of Nonsteroidal Anti-Inflammatory Drugs, Pharmaceutics. (2024) 16, no. 3, 10.3390/pharmaceutics16030382. PMC10975754 38543277

[39] Park C. , Meghani N. M. , Shin Y. et al., Investigation of Crystallization and Salt Formation of Poorly Water-Soluble Telmisartan for Enhanced Solubility, Pharmaceutics. (2019) 11, no. 3, 10.3390/pharmaceutics11030102, 2-s2.0-85064333882. PMC6470926 30823389

[40] Us Food and Drug Administration (Fda) , Draft Guidance on Buspirone Hydrochloride: Oral Tablet, 2024, FDA, https://www.fda.gov/regulatory-information/search-fda-guidance-documents.

